# Gastroptosis: A Diagnosis Overlooked

**DOI:** 10.14309/crj.0000000000001433

**Published:** 2024-07-11

**Authors:** Fady El Tom, Batoul Hammoud, Rodrigue Chemaly, Rajaa Chatila

**Affiliations:** 1 Department of Internal Medicine, Gilbert and Rose-Marie Chagoury School of Medicine, Lebanese American University, Byblos, Lebanon; 2 Department of Internal Medicine, Lebanese American University Medical Center Rizk Hospital, Beirut, Lebanon; 3 Department of Surgery, Gilbert and Rose-Marie Chagoury School of Medicine, Lebanese American University, Byblos, Lebanon; 4 Department of Surgery, Lebanese American University Medical Center Rizk Hospital, Beirut, Lebanon

## CASE REPORT

**Figure 1.**
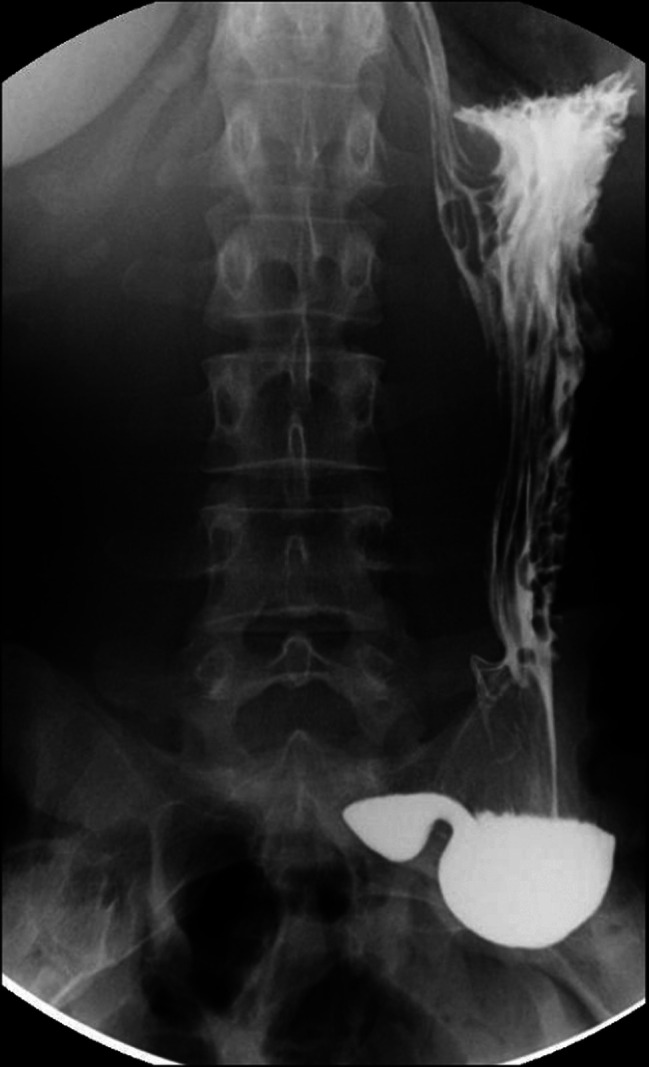
Upper gastrointestinal tract series results, which revealed the gastroptosis in our patient.

**Figure 2.**
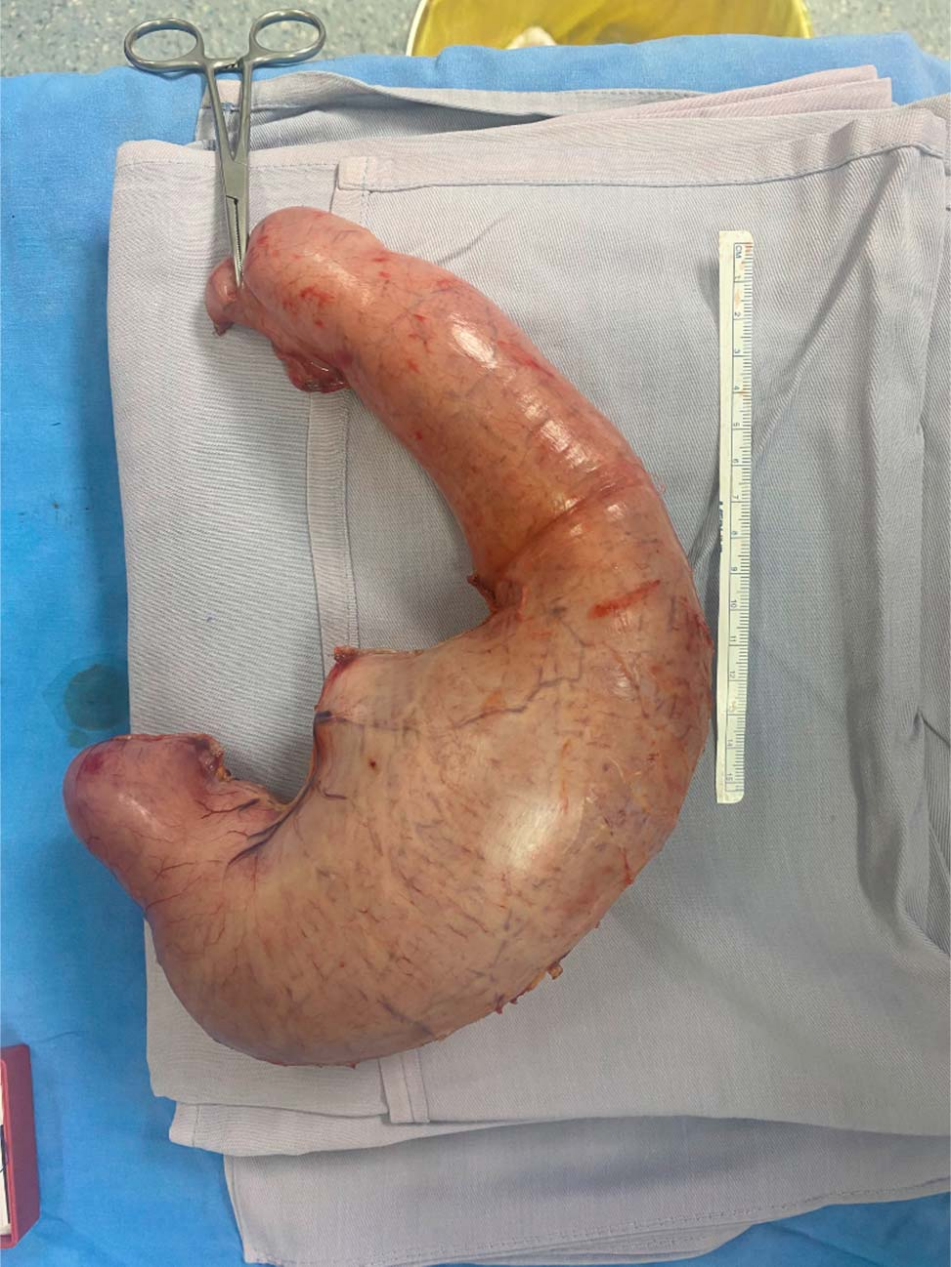
Extracted stomach upon gastrectomy with Roux-en-Y anastomosis.

**Figure 3.**
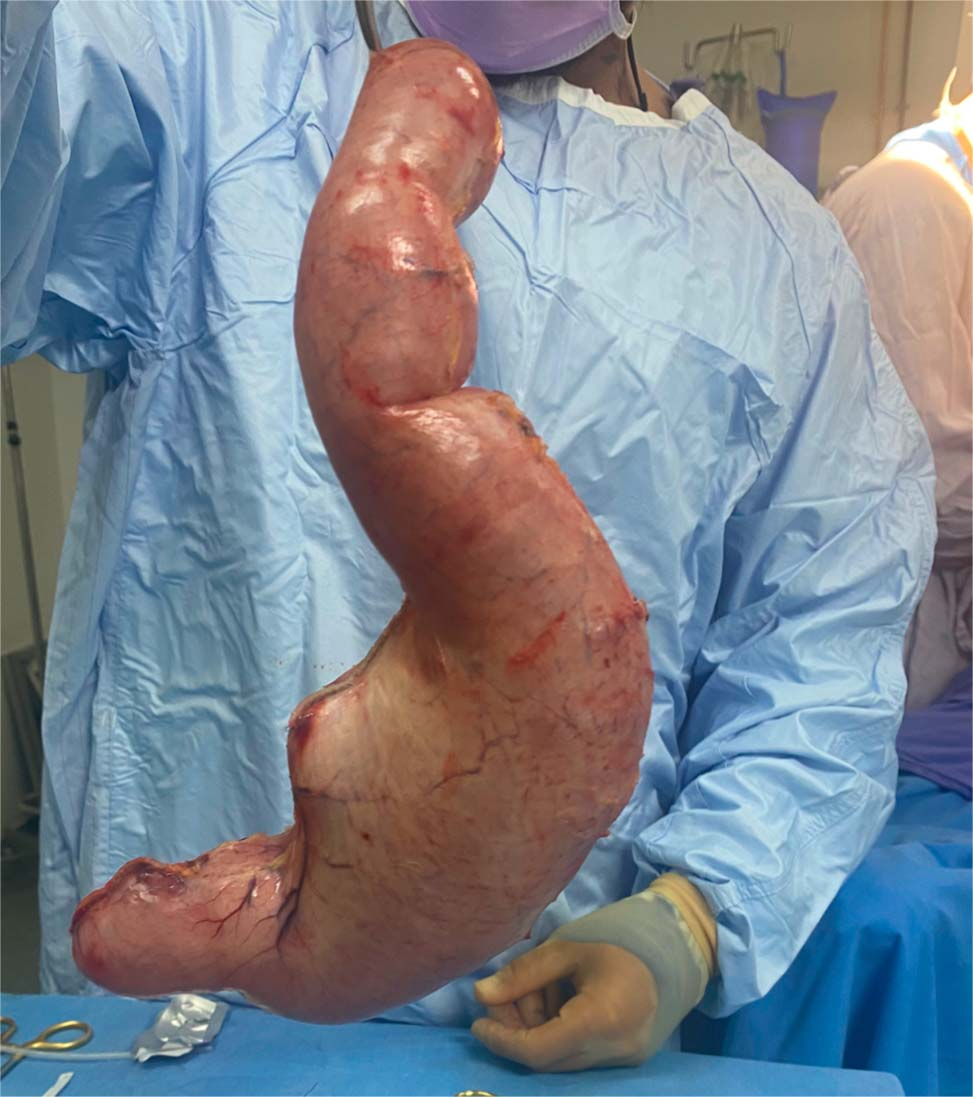
Extracted stomach held in vertical position to elicit ptosis.

We present the case of a 37-year-old woman with severe gastroptosis misdiagnosed as superior mesenteric artery syndrome, gastroparesis, and endometriosis. Within a month of cesarean section–assisted delivery of her second child, postprandial fullness, early satiety and nausea started and progressed over 5 years. Extensive workup including endoscopies, gastric emptying scintigraphy, abdominal computed tomography, and magnetic resonance imaging failed to yield a diagnosis. Despite proton pump inhibitors and various prokinetic drugs, her condition worsened leading to pelvic pain and weight loss. Alleviation of symptoms on reclining prompted us to request an upright upper gastrointestinal barium study revealing severe gastroptosis (Figure 1). The patient opted for a definitive treatment with laparoscopic Roux-en-Y gastrojejunostomy resulting in an uneventful recovery (Figures 2 and 3).

We hereby highlight the challenges of diagnosing gastroptosis, the downward displacement of the stomach.^1^ The nonspecific nature of symptoms makes accurate diagnosis elusive.^2^ It is important to inquire about positional alleviating factors and opt for upright imaging with oral contrast for definitive diagnosis.^3,4^ Surgical intervention is a viable option for refractory or advanced cases to enhance the patient's quality of life.^5^

Gastroptosis is likely underdiagnosed especially early in its course.^2^ We encourage clinicians to consider this condition in the differential diagnosis of dyspepsia, postprandial fullness, nausea, and early satiety.

## DISCLOSURES

Author contributions: F. El Tom and B. Hammoud: wrote, reviewed, and edited the manuscript. R. Chemaly and R. Chatila: supervised the writing of the original draft; reviewed and edited the manuscript.

Financial disclosure: None to report.

Informed consent was obtained for this case report.

## References

[1] Christianakis E Bouchra K Koliatou A Paschalidis N Filippou D. Gastroparesis associated with gastroptosis presenting as a lower abdominal bulking mass in a child: A case report. Cases J. 2009;2(11):184. 19946494 10.1186/1757-1626-2-184 PMC2783139

[2] Hall-Edwards JF. The diagnosis and treatment of gastroptosis. Br Med J. 1921;1(3150):698–700. 20770293 10.1136/bmj.1.3150.698 PMC2415035

[3] Sarangapani A Rasane S Kohli V Chandy G. Glenard’s disease. Arch Med Health Sci. 2016;4(1):153.

[4] Van Welie AJM Klein WM Th Draaisma JM. The clinical or radiographic diagnosis of gastroptosis: still relevant? Gastro Open J. 2017;2(1):14–9.

[5] Eve F. A clinical lecture on the surgical treatment of gastroptosis, with an account of five cases. Br Med J. 1906;1(2362):784–6. 20762603 10.1136/bmj.1.2362.784 PMC2381030

